# Risk Factors for Gastrointestinal Bleeding in Patients With Acute Myocardial Infarction: Multicenter Retrospective Cohort Study

**DOI:** 10.2196/67346

**Published:** 2025-01-30

**Authors:** Yanqi Kou, Shicai Ye, Yuan Tian, Ke Yang, Ling Qin, Zhe Huang, Botao Luo, Yanping Ha, Liping Zhan, Ruyin Ye, Yujie Huang, Qing Zhang, Kun He, Mouji Liang, Jieming Zheng, Haoyuan Huang, Chunyi Wu, Lei Ge, Yuping Yang

**Affiliations:** 1 Department of Gastroenterology Affiliated Hospital of Guangdong Medical University Zhanjiang China; 2 Department of Pathology Guangdong Medical University Zhanjiang China; 3 Department of Gastrointestinal Surgery Affiliated Hospital of Guangdong Medical University Zhanjiang China; 4 The First Affiliated Hospital, and College of Clinical Medicine of Henan University of Science and Technology Luoyang China; 5 Department of Colorectal Surgery Affiliated Hospital of Guangdong Medical University Zhanjiang China

**Keywords:** acute myocardial infarction, gastrointestinal bleeding, machine learning, in-hospital, prediction model

## Abstract

**Background:**

Gastrointestinal bleeding (GIB) is a severe and potentially life-threatening complication in patients with acute myocardial infarction (AMI), significantly affecting prognosis during hospitalization. Early identification of high-risk patients is essential to reduce complications, improve outcomes, and guide clinical decision-making.

**Objective:**

This study aimed to develop and validate a machine learning (ML)–based model for predicting in-hospital GIB in patients with AMI, identify key risk factors, and evaluate the clinical applicability of the model for risk stratification and decision support.

**Methods:**

A multicenter retrospective cohort study was conducted, including 1910 patients with AMI from the Affiliated Hospital of Guangdong Medical University (2005-2024). Patients were divided into training (n=1575) and testing (n=335) cohorts based on admission dates. For external validation, 1746 patients with AMI were included in the publicly available MIMIC-IV (Medical Information Mart for Intensive Care IV) database. Propensity score matching was adjusted for demographics, and the Boruta algorithm identified key predictors. A total of 7 ML algorithms—logistic regression, k-nearest neighbors, support vector machine, decision tree, random forest (RF), extreme gradient boosting, and neural networks—were trained using 10-fold cross-validation. The models were evaluated for the area under the receiver operating characteristic curve, accuracy, sensitivity, specificity, recall, *F*_1-_score, and decision curve analysis. Shapley additive explanations analysis ranked variable importance. Kaplan-Meier survival analysis evaluated the impact of GIB on short-term survival. Multivariate logistic regression assessed the relationship between coronary heart disease (CHD) and in-hospital GIB after adjusting for clinical variables.

**Results:**

The RF model outperformed other ML models, achieving an area under the receiver operating characteristic curve of 0.77 in the training cohort, 0.77 in the testing cohort, and 0.75 in the validation cohort. Key predictors included red blood cell count, hemoglobin, maximal myoglobin, hematocrit, CHD, and other variables, all of which were strongly associated with GIB risk. Decision curve analysis demonstrated the clinical use of the RF model for early risk stratification. Kaplan-Meier survival analysis showed no significant differences in 7- and 15-day survival rates between patients with AMI with and without GIB (*P*=.83 for 7-day survival and *P*=.87 for 15-day survival). Multivariate logistic regression showed that CHD was an independent risk factor for in-hospital GIB (odds ratio 2.79, 95% CI 2.09-3.74). Stratified analyses by sex, age, occupation, marital status, and other subgroups consistently showed that the association between CHD and GIB remained robust across all subgroups.

**Conclusions:**

The ML-based RF model provides a robust and clinically applicable tool for predicting in-hospital GIB in patients with AMI. By leveraging routinely available clinical and laboratory data, the model supports early risk stratification and personalized preventive strategies.

## Introduction

Acute myocardial infarction (AMI) remains one of the leading causes of mortality worldwide and is a significant contributor to long-term disability and health care resource use [1]. Nosocomial gastrointestinal bleeding (GIB), as a common complication of patients with AMI, may lead to a large number of morbidity and mortality and remains one of the key factors affecting the prognosis of patients [2,3]. The high incidence and profound clinical implications of GIB have made it a focal point of concern in the management of patients with AMI [4].

Existing studies have established a close association between AMI and the occurrence of GIB, particularly in patients undergoing antiplatelet or anticoagulant therapy [5]. In patients with AMI, the development of GIB not only complicates in-hospital treatment but also markedly increases mortality rates during hospitalization [6]. However, the precise mechanisms underlying GIB in patients with AMI have yet to be fully elucidated. Potential mechanisms include gastrointestinal mucosal injury induced by anticoagulant therapy, stress-related ulceration, and inflammatory responses [7,8]. In addition, underlying comorbidities such as coronary heart disease (CHD), diabetes mellitus, and hypertension may also play significant roles in the development of GIB [9,10].

Although several studies have investigated the incidence and clinical manifestations of GIB in patients with AMI, there is still a lack of systematic analysis regarding its potential risk factors [11]. Previous research has identified common risk factors for GIB in patients with AMI, including anemia, renal insufficiency, advanced age, and polypharmacy [12,13]. However, many of these studies rely on traditional univariate or multivariate statistical methods, which may be limited in addressing the complexity of multifactorial interactions [14]. With the increasing application of machine learning (ML) methods in medical research, there is an opportunity to leverage advanced algorithms for more precise prediction and identification of GIB risk factors in patients with AMI.

In this real-world study, we aimed to integrate ML methods with traditional statistical analysis to comprehensively evaluate the risk factors for in-hospital GIB in patients with AMI. The objectives were to develop and validate an effective predictive model and to assess the impact of GIB on short-term prognosis, ultimately providing more robust guidance for clinical practice.

## Methods

### Data Source

Participants were retrospectively included based on their admission to the Affiliated Hospital of Guangdong Medical University between January 2005 and June 2024. A total of 10,046 patients with AMI were initially screened for eligibility. Patients were excluded if they had a history of GIB within 1 month before admission or if more than 20% of their data were missing. After applying these criteria, 1910 eligible patients with AMI were included (more details in the patient selection flowchart in the *Results* section). The cohort was divided based on the admission date: patients admitted before January 1, 2023, were assigned to the training cohort (n=1575), and those admitted after this date were included in the testing cohort (n=335).

Data for external validation were extracted from the publicly available, single-center Medical Information Mart for Intensive Care IV (MIMIC-IV) database (version 3.0). A total of 1801 patients with AMI from MIMIC-IV were initially screened. After applying the same exclusion criteria as in the training cohort, a total of 1746 patients with AMI were included in the external validation cohort. The Beth Israel Deaconess Medical Center and the Massachusetts Institute of Technology have approved MIMIC-IV. The diagnosis of AMI was based on the *Fourth Universal Definition of Myocardial Infarction*, including both ST-segment elevation (ST elevation) and non–ST-segment elevation (non-ST elevation) AMI. GIB was defined as a clinically significant bleeding event diagnosed by a physician (manifesting as coffee-ground emesis, hematemesis, melena, or hematochezia) or the presence of blood in the upper or lower gastrointestinal tract identified during endoscopic evaluation.

### Data and Variables

The extracted variables included demographic characteristics, duration of hospital stay, discharge status, basic vital signs, and laboratory parameters. Cardiac injury biomarkers were recorded at their peak levels. Blood and biochemical test results were collected on the first day of admission. In cases where multiple test results were available for a specific variable, the first measurement was used in the analysis.

### Model Construction and Validation

The Boruta algorithm was used in this study to select significant variables from the training dataset. Boruta is a feature selection method based on random forests (RFs) that determines the importance of each variable by comparing its *z* score with that of its “shadow” counterparts [15]. During the algorithm’s execution, all real features were duplicated and randomly shuffled to generate *z* scores. If a real feature’s *z* score consistently exceeded the maximum *z* score of the shadow features across multiple independent tests, it was deemed important and included in subsequent ML model construction.

The important variables screened by the Boruta algorithm were incorporated into 7 different ML algorithms for model construction, including logistic regression (LR), k-nearest neighbor (KNN), support vector machine (SVM), decision tree (DCtree), RF, extreme gradient boosting (XGBoost), and artificial neural network (NNET) [16]. Hyperparameter tuning was conducted using grid search combined with 10-fold cross-validation to optimize the performance of all ML models. Key parameters such as the number of trees, maximum tree depth, and minimum samples per leaf were systematically varied during the grid search. To prevent overfitting, the dataset was divided into 10 folds, with 9 folds used for training and 1 fold for validation. This process was repeated across all folds to identify the combination of hyperparameters that yielded the highest area under the curve (AUC).

The model with the highest area under the receiver operating characteristic (ROC) curve was selected to determine model performance. Model discrimination was further assessed using sensitivity, specificity, recall, accuracy, and *F*_1_-score metrics. Decision curve analysis (DCA) was conducted to evaluate the clinical use of the models [17]. The best-performing model was subsequently used for further interpretive analysis. After training the model on the training cohort, all model parameters were fixed, and the model’s performance was further evaluated using the testing and validation cohort.

To better understand the decision-making process of the best-performing model, Shapley additive explanations (SHAP) were used [18]. Based on cooperative game theory’s Shapley values, SHAP rationally allocates contributions to the model’s output among individual input features. SHAP also reveals feature importance and visually displays the direction and magnitude of each feature’s contribution to the predictive outcome, thereby providing a deep understanding of the model’s decision-making process. In this study, SHAP analysis was instrumental in identifying risk factors associated with GIB in patients with AMI and evaluating their consistency and accuracy in clinical applications using the testing and validation cohort.

### Statistical Analysis

Multiple imputation was used to address missing data, including variables with a missing ratio of less than 20%. Continuous variables were expressed as medians and IQRs, while categorical variables were expressed as total numbers and percentages. Chi-square (*χ*^2^) tests, Wilcoxon rank-sum tests, or Fisher exact tests were used as appropriate. For survival analysis, Kaplan-Meier curves were used. Logistic regression analysis was also performed to evaluate the association between CHD and the occurrence of GIB in patients with AMI. Propensity score matching (PSM) was also conducted using the nearest neighbor method with a 1:1 ratio without replacement and a caliper width of 0.15. The analyses were performed using R software (R Foundation for Statistical Computing; version 4.2.2). Statistical significance was determined by a 2-tailed *P* value of less than .05.

### Ethical Considerations

This study was approved by the Institutional Review Board of the Affiliated Hospital of Guangdong Medical University (approval PJKT2024-176). The study used deidentified, retrospective clinical data, and informed consent was waived as the original consent obtained during initial data collection included provisions for secondary analysis. All patient data were anonymized, and confidentiality was strictly maintained. For the external validation cohort, database access was granted upon the completion of an online course and exam. As this portion of the study used a publicly accessible, deidentified database, informed consent was not required. Both data sources complied with all applicable ethical guidelines and privacy protection standards. The data from the training and testing cohorts were anonymized, eliminating the need for informed consent. In addition, the health information obtained from the MIMIC-IV database was deidentified, and therefore, patient consent was not required.

## Results

### Comparative Analysis of Clinical Profiles in Patients With AMI With and Without In-Hospital GIB

**Figure 1:**
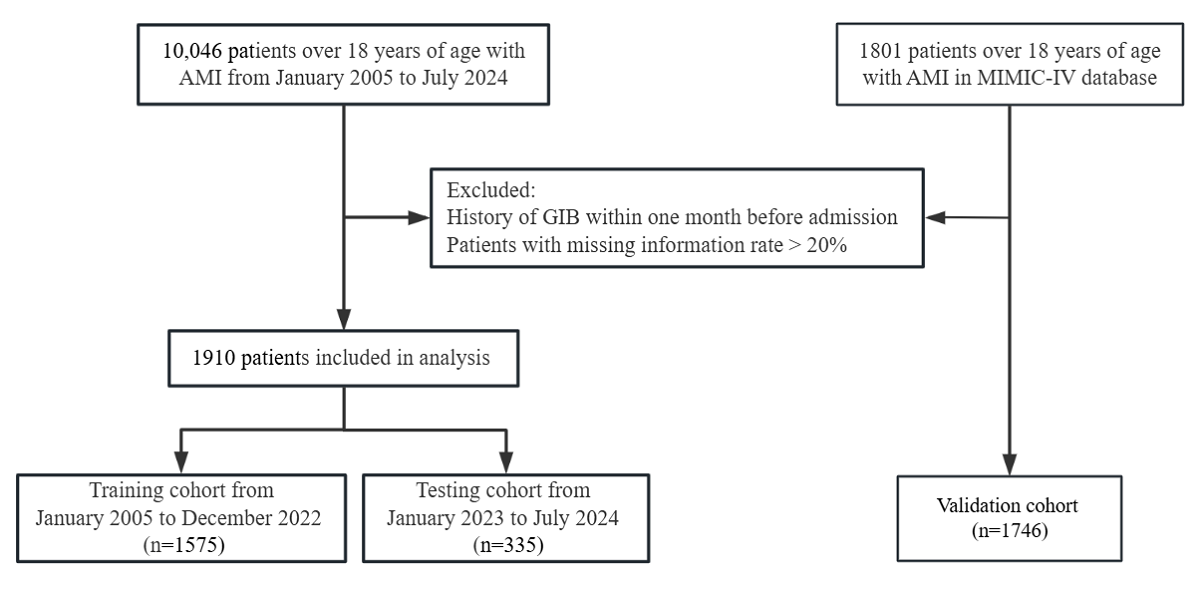
Patient selection flowchart for the training, testing, and validation cohorts. A total of 10,046 local patients and 1801 patients from the MIMIC-IV database were screened. After applying exclusion criteria, 1910 patients were included in the local cohort and 1746 patients in the external validation cohort. AMI: acute myocardial infarction; GIB: gastrointestinal bleeding; MIMIC-IV: Medical Information Mart for Intensive Care IV.

A total of 1575 patients with AMI were included in the training cohort (Figure 1), of whom 775 experienced in-hospital GIB. To minimize potential confounding factors, PSM was performed on variables such as sex, occupation, blood type, marital status, age, and length of hospital stay, achieving a balanced distribution of these selected characteristics between the groups. Table 1 compares the baseline characteristics before and after matching. After PSM, no statistically significant differences were observed between the groups for the matched variables (*P*>.05), indicating that the matching process effectively reduced confounding bias.

**Table 1:** Baseline demographic and clinical characteristics of patients with acute myocardial infarction with and without GIBa, before and after propensity score matching (2005-2024, Guangdong Medical University Hospital).

Characteristics			Unmatched								Matched							
			No GIB (n=800)			GIB (n=775)		*P* value			No GIB (n=731)			GIB (n=731)			*P* value	
**Age (years), mean (SD)**			69 (13)			71 (12)		<.001			70 (13)			71 (12)			.19	
**In-hospital length of stay (days), mean (SD)**			11 (9)			11 (9)		.85			11 (10)			11 (9)			.95	
**Gender, n (%)**									.008									.68
	Male	543 (68)		573 (7)						524 (72)			531 (73)					
	Female	257 (32)		202 (26)						207 (28)			200 (27)					
**Marriage status, n (%)**									.15									.66
	Married	758 (95)		721 (93)						689 (94)			685 (94)					
	Single, divorced, or windowed	42 (5)		54 (7)						42 (6)			46 (6)					
**Occupation, n (%)**									.65									.86
	Farmer	147 (18)		135 (17)						139 (19)			134 (18)					
	Employee	419 (52)		397 (51)						383 (52)			379 (52)					
	Retired or unemployed	234 (29)		243 (31)						209 (29)			218 (30)					
**ABO blood type,** **n (%)**									.29									.84
	A	202 (25)		189 (24)						183 (25)			170 (23)					
	O	301 (38)		318 (41)						287 (39)			299 (41)					
	B	250 (31)		214 (28)						215 (29)			213 (29)					
	AB	47 (6)		54 (7)						46 (6)			49 (7)					

^a^GIB: gastrointestinal bleeding.

The detailed demographic and baseline clinical characteristics of the PSM-adjusted training cohort are summarized in Table S1 in Multimedia Appendix 1. The results revealed significant differences in several laboratory parameters between patients with and without GIB. Specifically, patients with GIB exhibited significant differences in red blood cell count (RBC), hemoglobin, gamma-glutamyl transferase (GGT), total bilirubin (TBIL), direct bilirubin (DBIL), indirect bilirubin, albumin, total protein (TP), calcium, chloride, phosphorus, and N-terminal pro-brain natriuretic peptide maximum (NT-proBNP max), suggesting distinct clinical profiles related to anemia, cardiovascular status, liver function, electrolyte imbalance, and coagulation function.

The analysis suggests that patients with AMI who experience in-hospital GIB may have distinct clinical profiles, particularly in terms of anemia, cardiovascular health, liver function, electrolyte balance, and coagulation, which could inform more targeted management strategies.

### Key Predictor Identification Using Boruta Algorithm for In-Hospital GIB Risk in Patients With AMI

**Figure 2:**
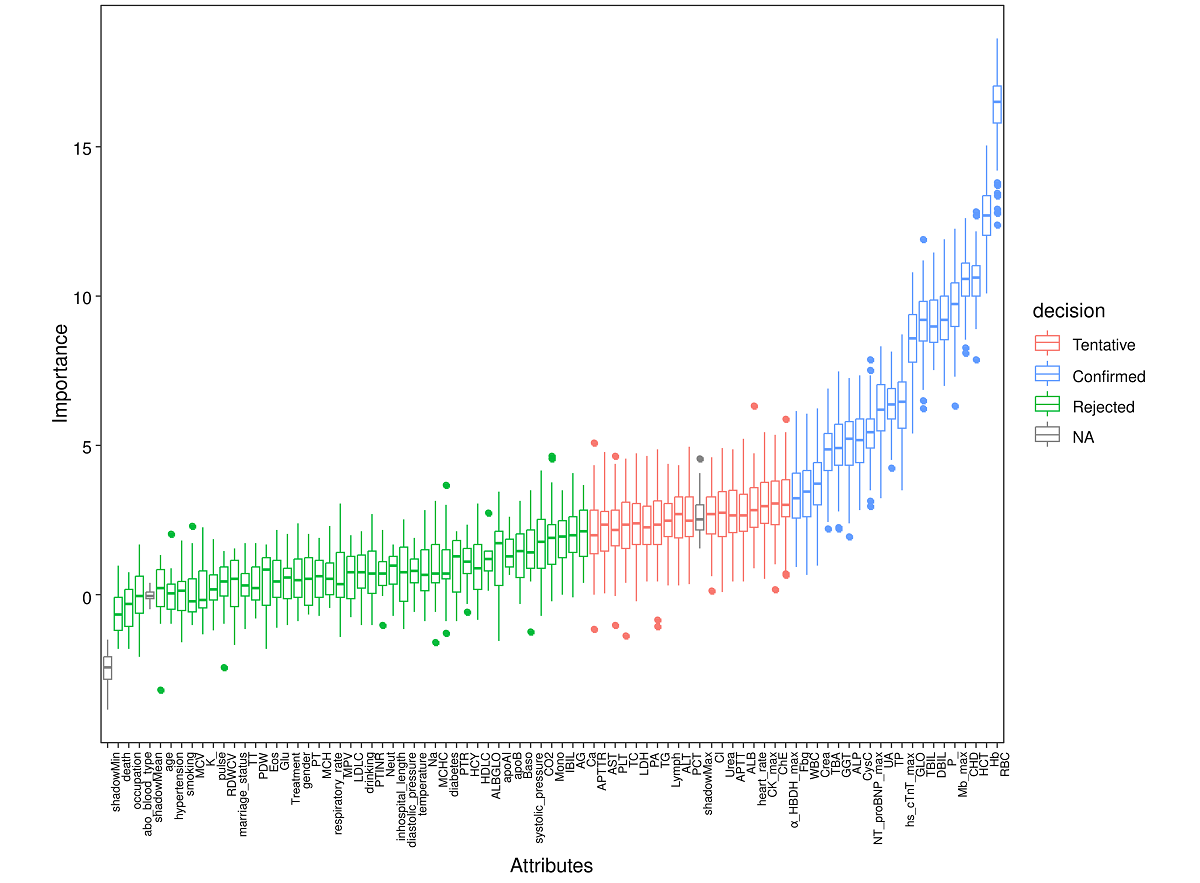
Feature selection for predicting gastrointestinal bleeding in patients with acute myocardial infarction using the Boruta algorithm. The horizontal axis displays the names of each variable, while the vertical axis displays the corresponding z scores of each variable. The box plot shows the z score of each variable during model calculation. The blue boxes represent important variables, the red represents tentative variables, and the green represents rejected variables. AG: anion gap; ALB: albumin; ALB/GLO: albumin-to-globulin ratio; ALP: alkaline phosphatase; ALT: alanine aminotransferase; Apob: apolipoprotein B; Apoai: apolipoprotein A-I; APTT: activated partial thromboplastin time; APTTR: activated partial thromboplastin time ratio; Baso: basophils; Ca: calcium; CHD: coronary heart disease; ChE: cholinesterase; Cl: chloride; Crea: creatinine; CysC: cystatin C; DBIL: direct bilirubin; Eos: eosinophils; Fbg: fibrinogen; Glu: glucose; GGT: gamma-glutamyl transferase; GLO: globulin; HDLC: high-density lipoprotein cholesterol; Hb: hemoglobin; HCT: hematocrit; HCY: homocysteine; IBIL: indirect bilirubin; K: potassium; LDH: lactate dehydrogenase; LDLC: low-density lipoprotein cholesterol; Lymph: lymphocytes; Mb max: myoglobin maximum; MCH: mean corpuscular hemoglobin; MCHC: mean corpuscular hemoglobin concentration; MCV: mean corpuscular volume; Mono: monocytes; Na: sodium; Neut: neutrophils; NT proBNP max: N-terminal pro b-type natriuretic peptide maximum; P: phosphorus; PA: prealbumin; PCT: plateletcrit; PLT: platelets; PTR: prothrombin time ratio; PT: prothrombin time; PTINR: prothrombin time international normalized ratio; RBC: red blood cell; RDWCV: red cell distribution width; TG: triglycerides; TC: total cholesterol; TBIL: total bilirubin; TBA: total bile acids; TT: thrombin time; UA: uric acid; WBC: white blood cell.

To identify key variables associated with GIB occurrence in patients with AMI, the Boruta algorithm was used for feature selection. Figure 2 illustrates the *z* scores of each variable, demonstrating how this analysis enhanced model optimization by concentrating on the most relevant features. A total of 20 variables were identified as important, including CHD, white blood cell count, RBC, hematocrit, hemoglobin, GGT, TBIL, DBIL, globulin, TP, total bile acids, creatinine, uric acid, phosphorus, alkaline phosphatase, cystatin C, fibrinogen, NT-proBNP max, maximal myoglobin, and maximal high-sensitivity cardiac troponin T, indicating their strong explanatory power in predicting GIB occurrence.

These variables were incorporated as primary predictors in the subsequent model construction and analysis, providing a solid foundation for developing predictive models.

### The RF Model Outperforms Other ML Models in Predicting In-Hospital GIB in Patients With AMI

**Figure 3:**
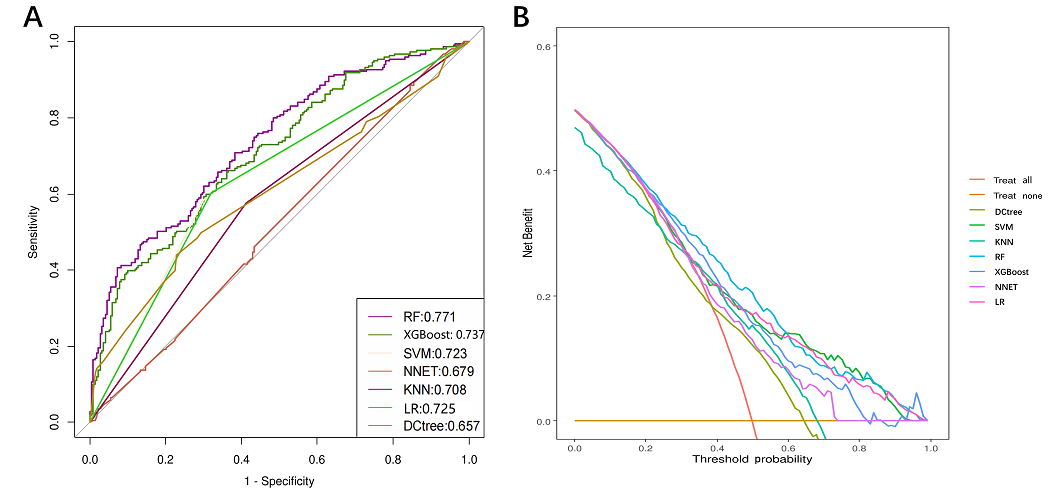
(A) Receiver operating characteristic curve and (B) decision curve analyses for 7 machine learning models predicting gastrointestinal bleeding in patients with acute myocardial infarction (training cohort). DCtree: decision tree; KNN: k-nearest neighbors; LR: logistic regression; NNET: neural network; RF: random forest; SVM: support vector machine; XGBoost: extreme gradient boosting.

The performance of 7 ML models in predicting GIB in patients with AMI was compared, and their ROC curves and DCA results were presented for the training cohort (Figure 3). The ROC curves (Figure 3A) demonstrate the discriminative ability of the various models, with the RF model achieving the highest AUC value (0.77, outperforming the others. The XGBoost (0.74) and SVM (0.72) models followed, while the DCtree model performed the poorest, with an AUC of 0.66. DCA (Figure 3B) assessed the clinical use of the models, further confirming that the RF model provided the highest net benefit across most threshold ranges, particularly within the intermediate range, highlighting its superiority in predicting GIB. The RF model demonstrated the best overall performance, with the highest AUC and net benefit, indicating its superior predictive capability for GIB. While other models, such as XGBoost and SVM, also performed well in certain scenarios, they were overall less effective than the RF model.

Further detailed performance metrics for each model, including sensitivity, specificity, recall, accuracy, and *F*_1_-score, are provided in Table 2. The RF model emerged as the top performer, with an accuracy of 0.66; an *F*_1_-score of 0.65; and balanced recall and specificity of 0.64 and 0.67, respectively, indicating its strong ability to distinguish between patients with and without GIB. The XGBoost model excelled in specificity, reaching 0.73, but its recall was lower at 0.58, resulting in an overall accuracy of 0.65 and an *F*_1_-score of 0.62. The SVM model achieved the highest recall (0.73) but suffered from low specificity (0.45), yielding an overall accuracy of 0.59 and an *F*_1_-score of 0.64. In contrast, the LR and KNN models exhibited similar performance, with accuracies of 0.61 and 0.60 and balanced recall and specificity around 0.60, reflecting moderate ability in distinguishing patients with GIB. The DCtree model reached a specificity of 0.73 but had lower recall (0.51), leading to an overall accuracy of 0.62 and an *F*_1_-score of 0.57. The NNET model performed the worst, with an accuracy of only 0.55 and an *F*_1_-score of 0.52, suggesting it may not be suitable for GIB prediction in this dataset.

**Table 2:** Performance comparison of machine learning models for predicting gastrointestinal bleeding in the training cohort.

Model	Accuracy	Sensitivity	Specificity	Recall	*F*_1-_score
RF^a^	0.66	0.64	0.67	0.64	0.65
XGBoost^b^	0.65	0.58	0.73	0.58	0.62
SVM^c^	0.59	0.73	0.45	0.73	0.64
NNET^d^	0.55	0.49	0.62	0.49	0.52
KNN^e^	0.60	0.60	0.60	0.60	0.60
LR^f^	0.61	0.61	0.60	0.61	0.61
DCtree^g^	0.62	0.51	0.73	0.51	0.57

^a^RF: random forest.

^b^XGBoost: extreme gradient boosting.

^c^SVM: support vector machine.

^d^NNET: neural network.

^e^KNN: k-nearest neighbors.

^f^LR: logistic regression.

^g^DCtree: decision tree.

Additional evaluations were conducted using both the testing and validation cohorts to assess the robustness of the models (Figures S1 and S2 in Multimedia Appendix 1; Tables S2 and S3 in Multimedia Appendix 1). The model’s performance was consistent, with an ROC value of 0.77 in the testing cohort and 0.75 in the validation cohort, demonstrating its generalizability and reliability across different datasets.

Overall, the RF model consistently outperformed other ML models in both predictive accuracy and clinical use, making it the most reliable tool for predicting in-hospital GIB in patients with AMI across multiple datasets.

### SHAP Analysis Quantifies Feature Contributions to In-Hospital GIB Risk in Patients With AMI

**Figure 4:**
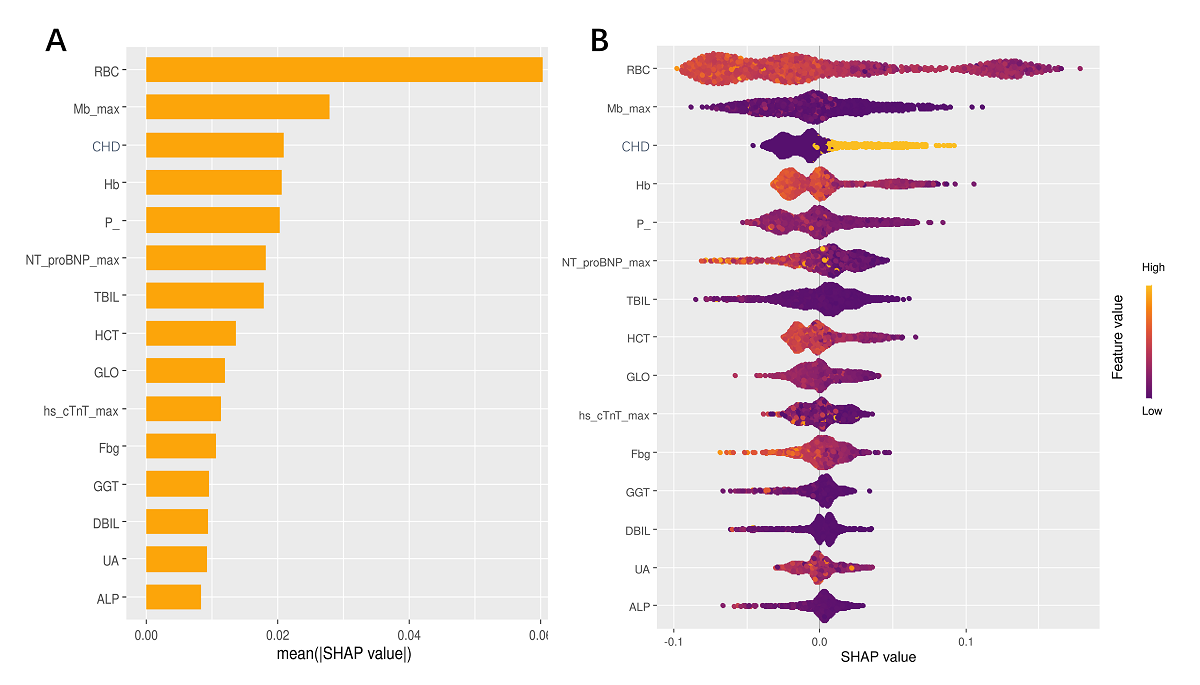
(A) SHAP (Shapley additive explanations) analysis of the top-15 predictors for gastrointestinal bleeding (GIB) in patients with acute myocardial infarction (AMI) using a random forest model ranked by mean absolute SHAP value, and (B) the impact of SHAP values on the occurrence of GIB in patients with AMI. ALP: alkaline phosphatase; CHD: coronary heart disease; DBIL: direct bilirubin; Fbg: fibrinogen; GGT: gamma-glutamyl transferase; GLO: globulin; HCT: hematocrit; Hb: hemoglobin; Hs cTnT Max: high-sensitivity cardiac troponin T maximum; Mb max: myoglobin maximum; NT proBNP max: N-terminal pro b-type natriuretic peptide maximum; P: phosphorus; RBC: red blood cell; TBIL: total bilirubin; UA: uric acid.

The SHAP analysis was used to interpret the predictions of the best-performing model, the RF. SHAP analysis highlighted the impact of 15 key features on the GIB prediction model in patients with AMI, ranked by SHAP values (Figure 4A). Features with positive SHAP values were associated with higher predicted GIB risk, while negative SHAP values indicated a lower risk. Darker colors represent decreases, while lighter colors indicate increases in the respective parameters, with all predictive factors positively correlated with GIB. The SHAP analysis also emphasized the importance of specific variables and provided insights into their directional influence on the prediction outcomes. RBC exhibited the strongest predictive influence, with lower values significantly increasing the predicted risk, followed by myoglobin maximum, CHD, hemoglobin, phosphorous, and NT-proBNP max, which also contributed to the prediction. TBIL and hematocrit further demonstrated their significance in the model. Other influential features included globulin, highly sensitive cardiac troponin T maximum, fasting blood glucose, GGT, DBIL, uric acid, and alkaline phosphatase. With varying SHAP values, these features illustrated their multidimensional impact within the predictive model (Figure 4B).

These features provided insights into the driving factors for GIB occurrence in patients with AMI, playing a crucial role in GIB prediction and understanding the underlying mechanisms. Notably, the SHAP values for CHD history indicated that an increase in this variable significantly heightened the risk of GIB, suggesting that CHD may play a crucial role in the pathophysiological mechanisms underlying GIB.

### Kaplan-Meier Analysis Shows No Significant Impact of GIB on Short-Term Survival in Patients With AMI

To assess the impact of GIB on short-term survival in patients with AMI, a Kaplan-Meier survival analysis was performed. Figure S3 in Multimedia Appendix 1 shows the survival curves for patients with and without GIB at 7 and 15 days. The results indicated that while overall survival rates were lower in the GIB group compared to the non-GIB group, the differences did not reach statistical significance (*P*=.83 for 7-day survival and *P*=.87 for 15-day survival). This suggests that in the study population, the occurrence of GIB did not significantly affect short-term survival in patients with AMI.

These findings suggest that while GIB is associated with lower survival rates, it does not significantly influence short-term mortality in patients with AMI within the study population.

### CHD Identified as a Significant Predictor of In-Hospital GIB Risk in Patients With AMI

Multivariate logistic regression was used to analyze further the association between CHD and in-hospital GIB in patients with AMI. The results, presented in Table 3, showed a significant association between CHD and the occurrence of GIB during hospitalization. In the unadjusted crude model, CHD was significantly positively associated with GIB (odds ratio [OR] 2.88, 95% CI 2.29-3.63; *P*<.001). After PSM, the association remained significant, with the OR slightly reduced to 2.50 (95% CI 1.98-3.17; *P*<.001). Even after stepwise adjustment for additional confounding variables, including sex, occupation, ABO blood type, marital status, age, and length of hospital stay (Model 1), hypertension, diabetes, smoking, alcohol consumption, body temperature, pulse, heart rate, respiratory rate, blood pressure (Model 2), and all laboratory parameters (Model 3), the significant association between CHD and GIB persisted. The OR values remained consistent across the different models, all retaining statistical significance (*P*<.001), indicating that CHD is an important risk factor for GIB during hospitalization in patients with AMI.

**Figure 5:**
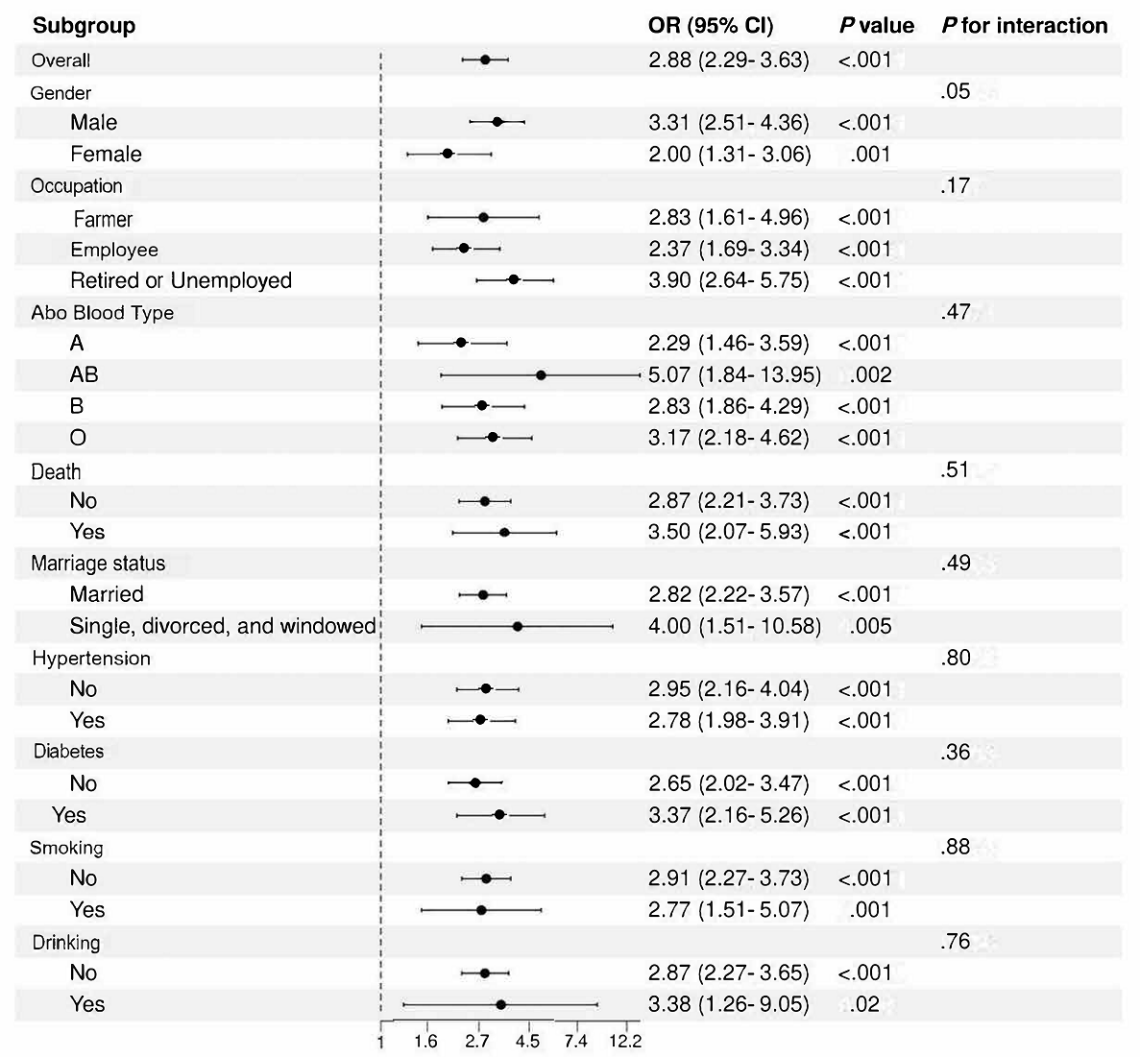
Subgroup analysis of coronary heart disease (CHD) impact on gastrointestinal bleeding (GIB) risk in patients with acute myocardial infarction (AMI). These results confirm that CHD is a consistent and significant risk factor for in-hospital GIB in patients with AMI, reinforcing its importance in risk stratification across diverse patient subgroups. OR: odds ratio.

Furthermore, Figure 5 presents the results of subgroup analysis and interaction tests conducted using the LR model to further explore the moderating effects of various characteristics on the relationship between CHD and GIB. Stratified analyses by sex, age, occupation, marital status, and other subgroups consistently showed that the association between CHD and GIB remained robust across all subgroups. This underscores the generalizability of CHD as a significant risk factor for GIB in patients with AMI, highlighting the importance of considering this risk factor in diverse patient populations.

**Table 3:** Association between coronary heart disease and risk of gastrointestinal bleeding in patients with acute myocardial infarction.

Variable	Overall			Propensity-score matched	
	OR^a^ (95% CI)	*P* value	OR (95% CI)		*P* value
Crude model^b^	2.88 (2.29-3.63)	<.001	2.50 (1.98-3.17)		<.001
Model 1^c^	2.74 (2.16-3.49)	<.001	2.56 (2.01-3.27)		<.001
Model 2^e^	2.70 (2.12-3.45)	<.001	2.54 (1.98-3.25)		<.001
Model 3^e^	2.92 (2.20-3.86)	<.001	2.79 (2.09-3.74)		<.001

^a^OR odds ratio.

^b^Crude model: No covariates were adjusted.

^c^Model 1: adjusted for gender, occupation, ABO blood type, marriage status, age, and in-hospital length of stay.

^d^Model 2: Model 1 and adjusted for hypertension, diabetes, smoking, drinking, temperature, pulse, heart rate, respiratory rate, diastolic pressure, and systolic pressure.

^e^Model 3: Model 2 and adjusted for all laboratory parameters.

## Discussion

### Principal Findings

This study systematically analyzes the risk factors associated with GIB in patients with AMI. Using the Boruta algorithm, significant variables were selected and compared across various ML models to construct an effective RF model for predicting GIB occurrence. This research not only enhances understanding of the mechanisms behind GIB in patients with AMI but also provides clinicians with a robust predictive tool aimed at reducing patient complications. In addition, Kaplan-Meier survival analysis was used to assess GIB’s impact on short-term survival rates and analyze the association between CHD and GIB, as well as its prognosis implications.

### Comparison With Previous Work

In comparison with existing literature, our study exhibits several unique advantages. First, we found that patients with GIB had significantly lower RBC, hemoglobin, and multiple liver function indicators compared to patients without GIB, aligning with previous research findings [19,20]. For instance, Martí et al [21] similarly identified anemia and hepatic dysfunction as pivotal risk factors for GIB in patients with AMI. Furthermore, Holster et al [22], through systematic review and meta-analysis, underscored the heightened risk of GIB associated with anticoagulant use, particularly among patients with underlying hepatic or hematologic conditions. This study further substantiates the significance of these factors through multivariable analysis and enhances prediction accuracy using ML methods. However, models from different studies exhibit variability in predicting GIB occurrence. For example, Chin et al [23] model heavily relies on traditional multivariable regression analysis, identifying significant risk factors such as advanced age, renal impairment, and anticoagulant use, albeit with limited predictive performance [23]. In contrast, our study’s adoption of the Boruta algorithm for feature selection and integration of the RF model not only enhances model robustness but also significantly improves predictive performance with an AUC value of 0.771. This underscores the capability of ML methods to offer precise risk prediction when handling complex, high-dimensional data.

Furthermore, through multivariable logistic regression analysis, this study further confirms a significant association between CHD and GIB occurrence, which persists even after adjusting for various confounding factors. Consistent with previous research, this suggests that patients with CHD may face increased GIB risk due to heightened inflammatory response and the necessity for anticoagulant therapy [24]. Subgroup analysis underscores the consistent impact of CHD across different patient demographics, emphasizing the need for heightened management attention, particularly in administering antiplatelet and anticoagulant therapies, which may necessitate stricter monitoring and personalized adjustments [25,26]. Kaplan-Meier survival analysis results indicate that although GIB is a common complication in patients with AMI, it does not significantly impact short-term survival rates. This finding aligns with the observations by Nikolsky et al [27] in patients after coronary artery bypass graft, suggesting minimal short-term prognosis effects among actively treated patients [27]. However, this does not negate the potential long-term impacts of GIB on patient outcomes, warranting further investigation.

### Strengths and Limitations

This study represents an innovative application of ML techniques to predict GIB in patients with AMI, integrating both clinical and laboratory data. The multicenter design, coupled with external validation using the MIMIC-IV database, enhances the generalizability of the findings across diverse patient populations. Robust validation methods, including PSM and the use of independent datasets, further strengthen the reliability of the predictive models and mitigate potential biases.

This study has several limitations that should be acknowledged. First, although it used a multicenter retrospective cohort design, the sample size may still be insufficient to fully capture the variability and complexity of GIB in patients with AMI. External validation using the MIMIC-IV database partially mitigates this limitation by increasing the generalizability of the findings. However, larger, multicenter prospective studies are needed to further validate these results across diverse populations. Second, some important variables, such as dietary habits and medication adherence, were not included in the analysis. These unmeasured confounding factors could influence the occurrence of GIB and limit the comprehensiveness and clinical applicability of the model. Incorporating additional predictors, such as dietary habits, medication adherence, and genetic markers, in future studies could enhance the model’s accuracy and provide a more holistic understanding of GIB risk. Third, the retrospective nature of the study introduces inherent limitations, including potential selection bias and reliance on existing medical records, which may include missing or incomplete data. In addition, in datasets where the limit of detection is an important factor, alternative imputation methods, such as minimal imputation, may provide more accurate processing of missing data. While this is not the main focus of this study, future studies could explore advanced imputation techniques tailored to specific missing mechanisms to improve the robustness of predictive models. To address this, PSM was applied to minimize confounding factors, and multiple imputation techniques were used to handle missing data. Despite these efforts, prospective studies with real-time clinical data collection would provide stronger evidence, reduce bias, and further validate the findings. In addition, leveraging advanced ML techniques and incorporating real-time clinical data may improve the model’s predictive performance and facilitate early intervention for high-risk patients.

### Conclusion

In summary, ML models, particularly the RF model, offer reliable tools for predicting GIB in patients with AMI and lay the groundwork for future research into its pathophysiology and prevention.

## Appendix

Multimedia Appendix 1 Risk factors for gastrointestinal bleeding (GIB) in patients with acute myocardial infarction (AMI).

## Abbreviations

AMI: acute myocardial infarction
AUC: area under the curve
CHD: coronary heart disease
DBIL: direct bilirubin
DCA: decision curve analysis
DCtree: decision tree
GGT: gamma-glutamyl transferase
GIB: gastrointestinal bleeding
KNN: k-nearest neighbors
LR: logistic regression
MIMIC-IV: Medical Information Mart for Intensive Care IV
ML: machine learning
NNET: neural network
NT-proBNP max: N-terminal pro-brain natriuretic peptide maximum
OR: odds ratio
PSM: propensity score matching
RBC: red blood cell count
RF: random forest
ROC: receiver operating characteristic
SHAP: Shapley additive explanations
SVM: support vector machine
TBIL: total bilirubin
TP: total protein
XGBoost: extreme gradient boosting

## Data Availability

The datasets generated during and/or analyzed during this study are available from the corresponding author on reasonable request.
