# Supplementary material for: Risk Factors for Gastrointestinal Bleeding in Patients With Acute Myocardial Infarction: Multicenter Retrospective Cohort Study
Source: J Med Internet Res. 2025 Jan 30;27:e67346. doi: 10.2196/67346 (PMC11826945; doi:10.2196/67346)
Supplement: Multimedia Appendix 1 [file jmir_v27i1e67346_app1.docx]

Supplementary materials


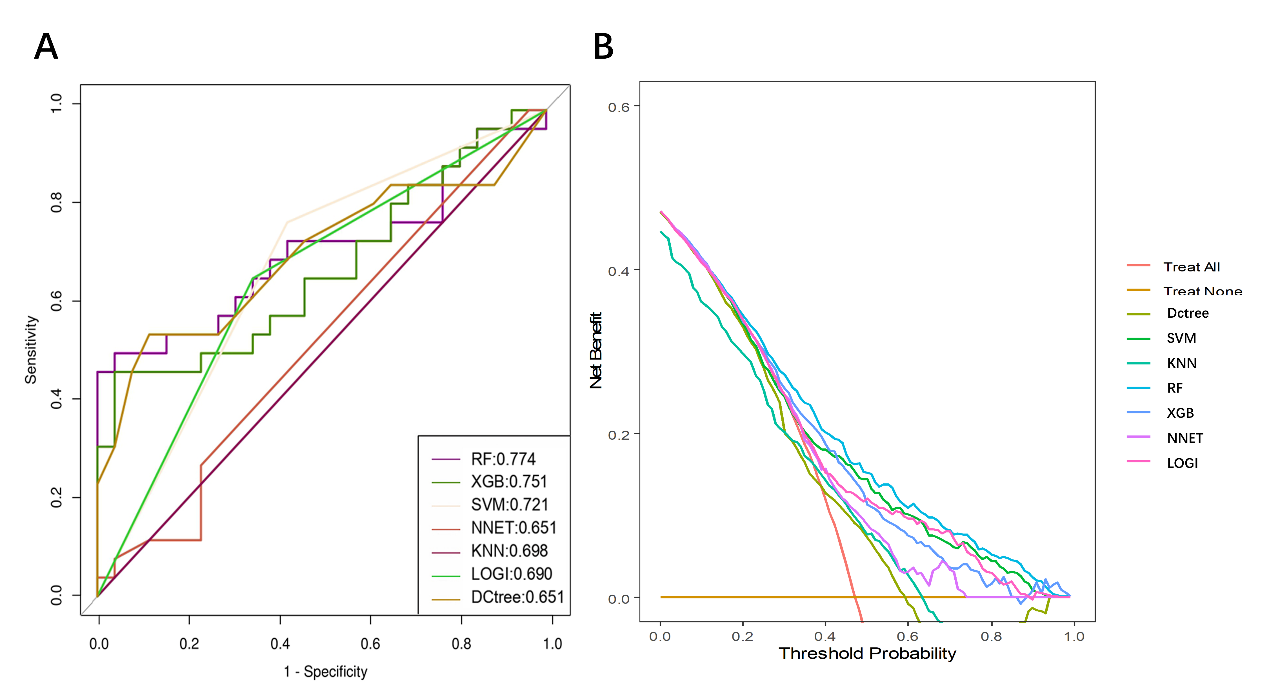


Figure S1. Receiver operating characteristic curve (A) and decision curve analyses (B) of the seven models in testing cohort.


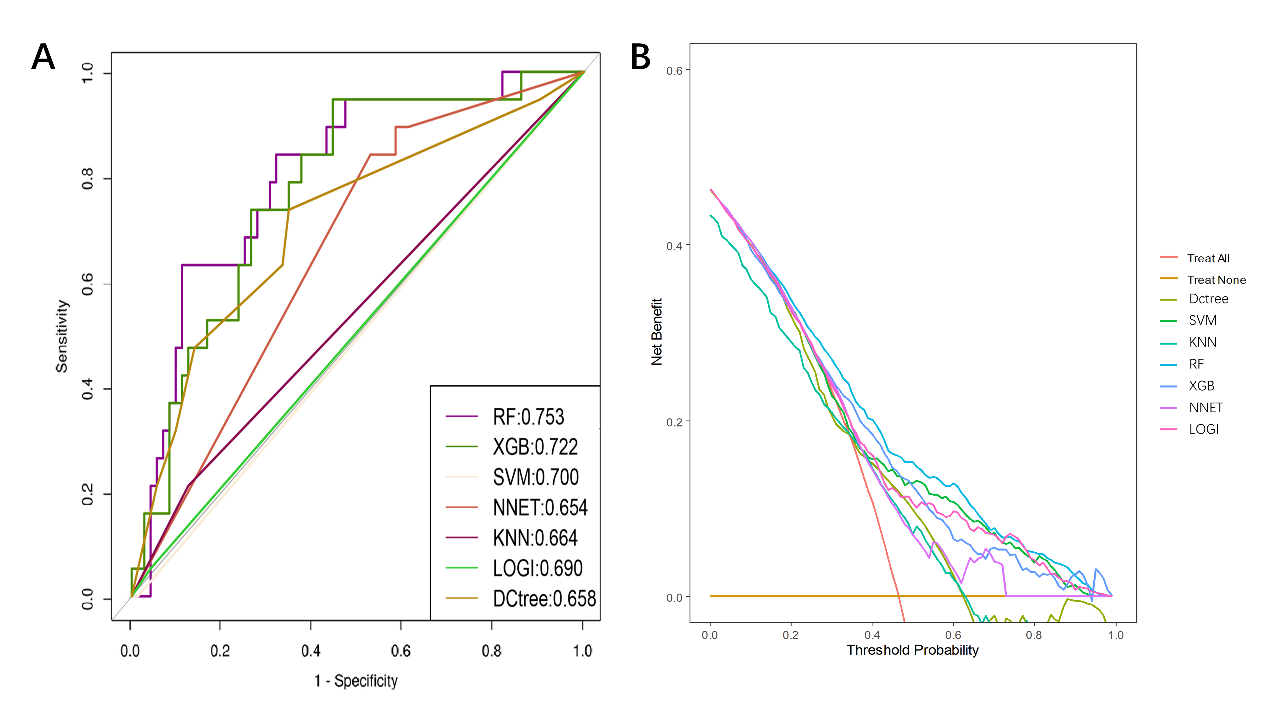


Figure S2. Receiver operating characteristic curve (A) and decision curve analyses (B) of the seven models in validation cohort.


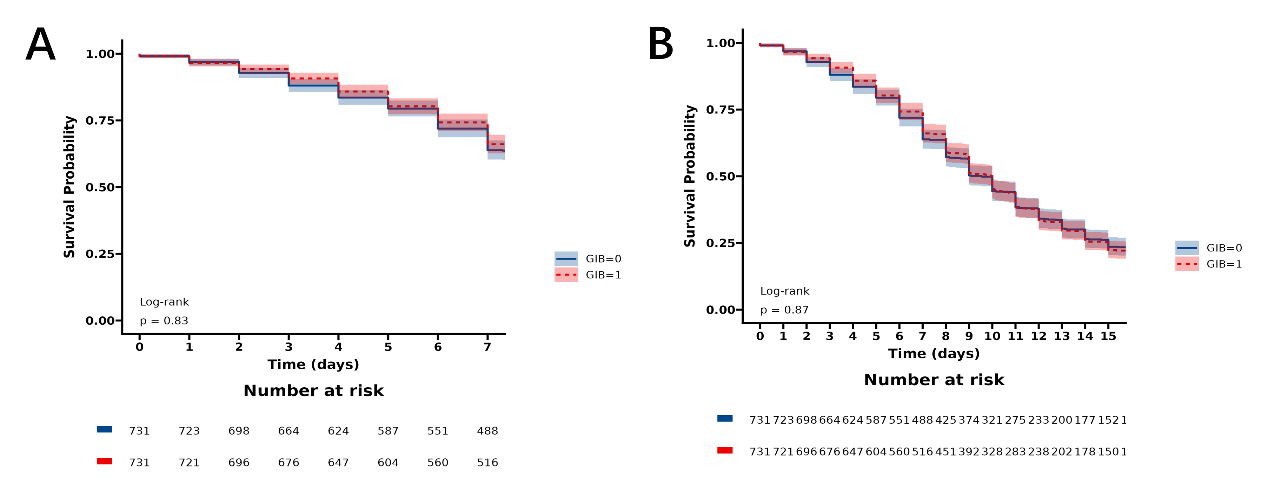


Figure S3. Kaplan-Meier estimates 7- and15- day survival in AMI Patients with GIB.

Table S1. Demographic and baseline characteristics of acute myocardial infarction patients in the training cohort (2005–2024, Guangdong Medical University Hospital).

| Variables | Total (n = 1462) | Non-GIB (n = 731) | GIB (n = 731) | *P* |
| --- | --- | --- | --- | --- |
|  |  |  |  |  |
| Age, M (IQR) | 72.00 (62.00, 79.00) | 71.00 (61.50, 79.00) | 72.00 (63.00, 79.00) | 0.190 |
| Inhospital Length, M (IQR) | 9.00 (6.00, 13.00) | 9.00 (6.00, 13.00) | 9.00 (6.00, 13.00) | 0.403 |
| Temperature, M (IQR) | 36.50 (36.50, 36.70) | 36.50 (36.50, 36.72) | 36.50 (36.50, 36.70) | 0.951 |
| Pulse, M (IQR) | 82.00 (72.00, 94.00) | 82.00 (73.00, 95.00) | 82.00 (72.00, 93.02) | 0.674 |
| Heart Rate, M (IQR) | 81.00 (74.28, 93.00) | 80.00 (74.00, 92.35) | 81.90 (75.00, 93.77) | 0.170 |
| Respiratory Rate, M (IQR) | 20.00 (20.00, 20.00) | 20.00 (19.93, 20.00) | 20.00 (20.00, 20.11) | 0.355 |
| Diastolic Pressure, M (IQR) | 75.00 (64.00, 84.00) | 75.00 (65.00, 85.00) | 74.00 (64.00, 84.00) | 0.441 |
| Systolic Pressure, M (IQR) | 136.00 (120.00, 154.00) | 134.50 (119.00, 153.00) | 138.00 (120.00, 156.00) | 0.076 |
| WBC, M (IQR) | 9.30 (7.50, 12.60) | 9.10 (7.42, 12.28) | 9.59 (7.55, 12.70) | 0.133 |
| RBC, M (IQR) | 4.02 (3.26, 4.49) | 4.18 (3.66, 4.57) | 3.72 (2.86, 4.39) | <.001 |
| HCT, M (IQR) | 36.50 (29.13, 40.98) | 38.00 (32.35, 41.30) | 34.30 (25.65, 40.35) | <.001 |
| MCV, M (IQR) | 90.30 (86.30, 93.80) | 90.40 (86.70, 93.90) | 90.30 (85.90, 93.60) | 0.249 |
| MCHC, M (IQR) | 331.15 (323.00, 339.00) | 331.70 (324.00, 339.00) | 331.00 (322.45, 338.55) | 0.119 |
| MCH, M (IQR) | 30.00 (28.50, 31.30) | 30.10 (28.70, 31.30) | 30.00 (28.40, 31.25) | 0.200 |
| RDWCV, M (IQR) | 14.20 (13.30, 15.40) | 14.10 (13.20, 15.30) | 14.30 (13.40, 15.60) | 0.075 |
| Lymph, M (IQR) | 1.23 (0.78, 1.80) | 1.20 (0.71, 1.77) | 1.29 (0.83, 1.90) | 0.019 |
| Mono, M (IQR) | 0.63 (0.47, 0.89) | 0.62 (0.45, 0.87) | 0.66 (0.48, 0.90) | 0.125 |
| Neut, M (IQR) | 7.26 (5.00, 11.06) | 7.30 (4.86, 11.09) | 7.23 (5.11, 11.02) | 0.666 |
| Eos, M (IQR) | 0.05 (0.01, 0.15) | 0.05 (0.00, 0.15) | 0.06 (0.01, 0.14) | 0.487 |
| Baso, M (IQR) | 0.02 (0.00, 0.03) | 0.02 (0.00, 0.03) | 0.02 (0.00, 0.03) | 0.337 |
| Hb, M (IQR) | 120.00 (95.00, 136.00) | 126.00 (106.00, 138.00) | 113.00 (82.70, 133.10) | <.001 |
| PLT, M (IQR) | 212.00 (165.85, 263.00) | 207.00 (159.30, 255.60) | 217.90 (173.05, 269.85) | 0.004 |
| MPV, M (IQR) | 8.85 (7.80, 10.00) | 8.80 (7.80, 10.00) | 8.90 (7.80, 10.00) | 0.687 |
| PDW, M (IQR) | 16.40 (12.20, 17.00) | 16.40 (12.20, 17.00) | 16.40 (12.15, 16.90) | 0.723 |
| PCT, M (IQR) | 0.19 (0.15, 0.22) | 0.18 (0.15, 0.22) | 0.19 (0.15, 0.23) | 0.056 |
| ALT, M (IQR) | 24.60 (14.90, 48.51) | 25.60 (14.80, 56.88) | 24.00 (15.30, 44.30) | 0.161 |
| AST, M (IQR) | 33.70 (20.00, 107.27) | 33.20 (19.70, 120.41) | 33.80 (20.20, 95.50) | 0.486 |
| GGT, M (IQR) | 32.00 (20.00, 59.87) | 36.00 (20.00, 75.89) | 30.00 (19.90, 49.77) | <.001 |
| TBIL, M (IQR) | 11.50 (8.10, 16.00) | 12.60 (9.30, 17.65) | 10.50 (7.60, 14.50) | <.001 |
| DBIL, M (IQR) | 4.70 (3.40, 6.80) | 4.93 (3.60, 8.10) | 4.40 (3.20, 6.10) | <.001 |
| IBIL, M (IQR) | 6.60 (4.30, 9.80) | 7.00 (4.50, 10.55) | 6.10 (4.00, 9.06) | <.001 |
| ALB, M (IQR) | 35.90 (32.00, 39.40) | 36.32 (32.55, 39.89) | 35.40 (31.60, 38.90) | <.001 |
| GLO, M (IQR) | 26.26 (23.70, 28.70) | 26.68 (24.60, 29.00) | 25.60 (23.00, 28.41) | <.001 |
| TP, M (IQR) | 62.60 (57.62, 67.20) | 63.80 (59.30, 67.80) | 61.60 (56.10, 66.54) | <.001 |
| ALB/GLO, M (IQR) | 1.39 (1.20, 1.58) | 1.37 (1.19, 1.54) | 1.40 (1.20, 1.60) | 0.070 |
| PA, M (IQR) | 191.20 (137.55, 241.57) | 193.60 (132.62, 242.58) | 188.90 (142.35, 238.95) | 0.873 |
| TBA, M (IQR) | 3.70 (2.00, 6.80) | 4.30 (2.30, 8.05) | 3.29 (1.80, 5.95) | <.001 |
| Crea, M (IQR) | 96.00 (75.00, 153.95) | 95.00 (75.00, 161.00) | 97.00 (75.30, 147.05) | 0.896 |
| Urea, M (IQR) | 6.95 (5.20, 11.02) | 6.98 (5.20, 10.92) | 6.93 (5.12, 11.10) | 0.908 |
| UA, M (IQR) | 380.83 (313.08, 457.88) | 393.00 (316.15, 467.43) | 367.30 (312.06, 453.22) | 0.051 |
| TC, M (IQR) | 4.19 (3.34, 5.11) | 4.24 (3.40, 5.21) | 4.10 (3.28, 4.96) | 0.038 |
| TG, M (IQR) | 1.20 (0.88, 1.70) | 1.24 (0.90, 1.79) | 1.17 (0.87, 1.59) | 0.010 |
| HDLC, M (IQR) | 1.10 (0.86, 1.34) | 1.12 (0.85, 1.36) | 1.09 (0.87, 1.31) | 0.312 |
| LDLC, M (IQR) | 2.39 (1.75, 3.20) | 2.44 (1.76, 3.25) | 2.36 (1.73, 3.11) | 0.118 |
| Apoai, M (IQR) | 1.03 (0.84, 1.23) | 1.04 (0.83, 1.24) | 1.03 (0.85, 1.21) | 0.596 |
| Apob, M (IQR) | 0.86 (0.68, 1.04) | 0.88 (0.69, 1.06) | 0.84 (0.68, 1.01) | 0.029 |
| Ca, M (IQR) | 2.15 (2.03, 2.25) | 2.17 (2.04, 2.27) | 2.13 (2.01, 2.24) | <.001 |
| K, M (IQR) | 3.99 (3.66, 4.38) | 3.98 (3.66, 4.34) | 4.00 (3.67, 4.41) | 0.336 |
| Na, M (IQR) | 139.30 (136.20, 141.60) | 139.20 (136.00, 141.70) | 139.40 (136.30, 141.60) | 0.371 |
| Cl, M (IQR) | 103.20 (99.30, 106.30) | 102.50 (98.90, 105.90) | 103.70 (100.20, 106.65) | <.001 |
| P, M (IQR) | 1.07 (0.94, 1.25) | 1.09 (0.97, 1.27) | 1.03 (0.91, 1.23) | <.001 |
| LDH, M (IQR) | 266.00 (188.22, 454.45) | 268.70 (192.55, 473.60) | 263.00 (186.00, 431.16) | 0.441 |
| ALP, M (IQR) | 73.00 (58.00, 94.07) | 77.00 (60.25, 100.65) | 70.00 (56.00, 88.85) | <.001 |
| ChE, M (IQR) | 6.00 (4.40, 7.67) | 6.23 (4.61, 7.82) | 5.77 (4.20, 7.53) | 0.009 |
| HCY, M (IQR) | 13.08 (10.40, 16.90) | 13.50 (10.80, 17.35) | 12.60 (10.13, 16.45) | 0.012 |
| CysC, M (IQR) | 1.10 (0.86, 1.55) | 1.13 (0.87, 1.64) | 1.08 (0.85, 1.47) | 0.030 |
| CO2, M (IQR) | 21.10 (18.20, 23.50) | 21.10 (18.10, 23.60) | 21.20 (18.20, 23.30) | 0.587 |
| AG, M (IQR) | 14.70 (12.00, 17.70) | 15.10 (12.20, 18.50) | 14.30 (11.80, 17.05) | 0.002 |
| Glu, M (IQR) | 7.19 (5.71, 9.35) | 7.17 (5.71, 9.12) | 7.22 (5.69, 9.64) | 0.835 |
| TT, M (IQR) | 17.50 (16.10, 19.30) | 17.70 (16.20, 19.35) | 17.20 (16.00, 19.30) | 0.044 |
| PT, M (IQR) | 12.95 (11.80, 14.50) | 12.90 (11.90, 14.70) | 13.00 (11.80, 14.20) | 0.300 |
| PTR, M (IQR) | 1.08 (0.98, 1.20) | 1.07 (0.99, 1.22) | 1.08 (0.98, 1.18) | 0.483 |
| APTT, M (IQR) | 34.00 (29.10, 39.70) | 34.50 (29.60, 40.35) | 33.30 (28.80, 39.10) | 0.019 |
| APTTR, M (IQR) | 1.08 (0.97, 1.24) | 1.09 (0.98, 1.25) | 1.08 (0.96, 1.22) | 0.073 |
| PTINR, M (IQR) | 1.05 (0.97, 1.17) | 1.05 (0.97, 1.20) | 1.05 (0.97, 1.16) | 0.414 |
| Fbg, M (IQR) | 3.66 (2.84, 4.55) | 3.79 (2.96, 4.69) | 3.50 (2.74, 4.44) | <.001 |
| NT proBNP max, M (IQR) | 4886.87 (1923.76, 10360.09) | 5631.22 (2324.87, 12390.27) | 4155.00 (1657.77, 8820.66) | <.001 |
| Mb max, M (IQR) | 217.05 (80.07, 471.10) | 261.26 (101.35, 513.89) | 157.87 (71.55, 422.50) | <.001 |
| CK max, M (IQR) | 238.05 (95.00, 865.13) | 211.85 (93.00, 846.80) | 263.00 (97.10, 898.00) | 0.133 |
| Hs cTnT Max, M (IQR) | 0.79 (0.19, 1.85) | 1.02 (0.20, 1.93) | 0.59 (0.19, 1.59) | <.001 |
| α HBDH max, M (IQR) | 273.31 (179.04, 432.65) | 281.51 (183.20, 439.35) | 265.00 (176.78, 424.66) | 0.119 |
| Treatment, n(%) |  |  |  | 0.058 |
| PCI | 580 (39.67) | 266 (36.39) | 314 (42.95) |  |
| Thrombolysis | 67 (4.58) | 37 (5.06) | 30 (4.10) |  |
| Gender, n(%) |  |  |  | 0.683 |
| Male | 1055 (72.16) | 524 (71.68) | 531 (72.64) |  |
| Female | 407 (27.84) | 207 (28.32) | 200 (27.36) |  |
| Occupation, n(%) |  |  |  | 0.860 |
| Farmer | 273 (18.67) | 139 (19.02) | 134 (18.33) |  |
| Employee | 762 (52.12) | 383 (52.39) | 379 (51.85) |  |
| Retired/Unemployed | 427 (29.21) | 209 (28.59) | 218 (29.82) |  |
| Abo Blood Type, n(%) |  |  |  | 0.843 |
| A | 353 (24.15) | 183 (25.03) | 170 (23.26) |  |
| AB | 95 (6.50) | 46 (6.29) | 49 (6.70) |  |
| B | 428 (29.27) | 215 (29.41) | 213 (29.14) |  |
| O | 586 (40.08) | 287 (39.26) | 299 (40.90) |  |
| Death, n(%) |  |  |  | 0.662 |
| No | 1240 (84.82) | 617 (84.40) | 623 (85.23) |  |
| Yes | 222 (15.18) | 114 (15.60) | 108 (14.77) |  |
| Marriage Status, n(%) |  |  |  | 0.660 |
| Married | 1374 (93.98) | 689 (94.25) | 685 (93.71) |  |
| Single, divorced, and windowed | 88 (6.02) | 42 (5.75) | 46 (6.29) |  |
| Hypertension, n(%) |  |  |  | <.001 |
| No | 773 (52.87) | 419 (57.32) | 354 (48.43) |  |
| Yes | 689 (47.13) | 312 (42.68) | 377 (51.57) |  |
| CHD, n(%) |  |  |  | <.001 |
| No | 1040 (71.14) | 587 (80.30) | 453 (61.97) |  |
| Yes | 422 (28.86) | 144 (19.70) | 278 (38.03) |  |
| Diabetes, n(%) |  |  |  | 0.001 |
| No | 1073 (73.39) | 564 (77.15) | 509 (69.63) |  |
| Yes | 389 (26.61) | 167 (22.85) | 222 (30.37) |  |
| Smoking, n(%) |  |  |  | 0.511 |
| No | 1241 (84.88) | 625 (85.50) | 616 (84.27) |  |
| Yes | 221 (15.12) | 106 (14.50) | 115 (15.73) |  |
| Drinking, n(%) |  |  |  | <.001 |
| No | 1355 (92.68) | 694 (94.94) | 661 (90.42) |  |
| Yes | 107 (7.32) | 37 (5.06) | 70 (9.58) |  |

Abbreviations: GIB, gastrointestinal bleeding; M, median; IQR, interquartile range; ALB/GLO, albumin-to-globulin ratio; WBC, white blood cell; RBC, red blood cell; HCT, hematocrit; MCV, mean corpuscular volume; MCHC, mean corpuscular hemoglobin concentration; MCH, mean corpuscular hemoglobin; RDWCV, red cell distribution width; Lymph, lymphocytes; Mono, monocytes; Neut, neutrophils; Eos, eosinophils; Baso, basophils; Hb, hemoglobin; PLT, platelets; MPV, mean platelet volume; PDW, platelet distribution width; PCT, plateletcrit; ALT, alanine aminotransferase; AST, aspartate aminotransferase; GGT, gamma-glutamyl transferase; TBIL, total bilirubin; DBIL, direct bilirubin; IBIL, indirect bilirubin; ALB, albumin; GLO, globulin; TP, total protein; PA, prealbumin; TBA, total bile acids; Crea, creatinine; UA, uric acid; TC, total cholesterol; TG, triglycerides; HDLC, high-density lipoprotein cholesterol; LDLC, low-density lipoprotein cholesterol; Apoai, apolipoprotein A-I; Apob, apolipoprotein B; Ca, calcium; K, potassium; Na, sodium; Cl, chloride; P, phosphorus; LDH, lactate dehydrogenase; ALP, alkaline phosphatase; ChE, cholinesterase; HCY, homocysteine; CysC, cystatin C; CO2, carbon dioxide; AG, anion gap; Glu, glucose; TT, thrombin time; PT, prothrombin time; PTR, prothrombin time ratio; APTT, activated partial thromboplastin time; APTTR, activated partial thromboplastin time ratio; PTINR, prothrombin time international normalized ratio; Fbg, fibrinogen; NT proBNP max, N-terminal pro b-type natriuretic peptide maximum; Mb max, myoglobin maximum; CK max, creatine kinase maximum; Hs cTnT Max, high-sensitivity cardiac troponin T maximum; α HBDH max, alpha-hydroxybutyrate dehydrogenase maximum; PCI, percutaneous coronary intervention; CHD, coronary heart disease.

Table S2. Comparison of testing cohort results of the machine learning models

| Model | Accuracy | Sensitivity | Specificity | Recall | F1score |
| --- | --- | --- | --- | --- | --- |
| RF | 0.635 | 0.615 | 0.654 | 0.615 | 0.627 |
| XGB | 0.577 | 0.538 | 0.615 | 0.538 | 0.560 |
| SVM | 0.673 | 0.769 | 0.577 | 0.769 | 0.702 |
| NNET | 0.442 | 0.115 | 0.769 | 0.115 | 0.171 |
| KNN | 0.500 | 0.538 | 0.462 | 0.538 | 0.519 |
| LOGI | 0.654 | 0.654 | 0.654 | 0.654 | 0.654 |
| DCtree | 0.654 | 0.538 | 0.769 | 0.538 | 0.609 |

Abbreviations: RF, Random Forest; XGB, eXtreme Gradient Boosting; SVM, Support Vector Machine; NNET, Neural Network; KNN, K-Nearest Neighbors; LOGI, Logistic Regression; DCtree, Decision Tree.

Table S3. Comparison of validation cohort results of the machine learning models

| Model | Accuracy | Sensitivity | Specificity | Recall | F1score |
| --- | --- | --- | --- | --- | --- |
| RF | 0.658 | 0.684 | 0.632 | 0.684 | 0.667 |
| XGB | 0.605 | 0.684 | 0.526 | 0.684 | 0.634 |
| SVM | 0.658 | 0.624 | 0.632 | 0.624 | 0.667 |
| NNET | 0.632 | 0.684 | 0.579 | 0.684 | 0.650 |
| KNN | 0.579 | 0.421 | 0.737 | 0.421 | 0.500 |
| LOGI | 0.658 | 0.632 | 0.684 | 0.632 | 0.649 |
| DCtree | 0.602 | 0.632 | 0.633 | 0.632 | 0.612 |

Abbreviations: RF, Random Forest; XGB, eXtreme Gradient Boosting; SVM, Support Vector Machine; NNET, Neural Network; KNN, K-Nearest Neighbors; LOGI, Logistic Regression; DCtree, Decision Tree.
